# Site-directed mutagenesis of the quorum-sensing transcriptional regulator SinR affects the biosynthesis of menaquinone in *Bacillus subtilis*

**DOI:** 10.1186/s12934-021-01603-5

**Published:** 2021-06-07

**Authors:** Jing Wu, Wei Li, Shi-guang Zhao, Sen-he Qian, Zhou Wang, Meng-jie Zhou, Wen-song Hu, Jian Wang, Liu-xiu Hu, Yan Liu, Zheng-lian Xue

**Affiliations:** 1 College of Biology and Food Engineering, Anhui Polytechnic University, Wuhu, 241000 China; 2 Anhui Engineering Laboratory for Industrial Microbiology Molecular Breeding, Wuhu, 241000 China; 3 Wuhu Zhanghengchun Medicine CO., LTD, Wuhu, 241000 China

**Keywords:** *Bacillus subtilis*, Menaquinone, Transcriptional regulator, Site-directed mutagenesis, SinR

## Abstract

**Background:**

Menaquinone (MK-7) is a highly valuable vitamin K_2_ produced by *Bacillus subtilis*. Common static metabolic engineering approaches for promoting the production of MK-7 have been studied previously. However, these approaches caused an accumulation of toxic substances and reduced product yield. Hence, dynamic regulation by the quorum sensing (QS) system is a promising method for achieving a balance between product synthesis and cell growth.

**Results:**

In this study, the QS transcriptional regulator SinR, which plays a significant role in biofilm formation and MK production simultaneously, was selected, and its site-directed mutants were constructed. Among these mutants, *sinR* knock out strain (KO-SinR) increased the biofilm biomass by 2.8-fold compared to the wild-type. SinR^quad^ maximized the yield of MK-7 (102.56 ± 2.84 mg/L). To decipher the mechanism of how this mutant regulates MK-7 synthesis and to find additional potential regulators that enhance MK-7 synthesis, RNA-seq was used to analyze expression changes in the QS system, biofilm formation, and MK-7 synthesis pathway. The results showed that the expressions of *tapA*, *tasA* and *epsE* were up-regulated 9.79-, 0.95-, and 4.42-fold, respectively. Therefore, SinR^quad^ formed more wrinkly and smoother biofilms than BS168. The upregulated expressions of *glpF*, *glpk*, and *glpD* in this biofilm morphology facilitated the flow of glycerol through the biofilm. In addition, NADH dehydrogenases especially *sdhA*, *sdhB*, *sdhC* and *glpD*, increased 1.01-, 3.93-, 1.87-, and 1.11-fold, respectively. The increased expression levels of NADH dehydrogenases indicated that more electrons were produced for the electron transport system. Electrical hyperpolarization stimulated the synthesis of the electron transport chain components, such as cytochrome c and MK, to ensure the efficiency of electron transfer. Wrinkly and smooth biofilms formed a network of interconnected channels with a low resistance to liquid flow, which was beneficial for the uptake of glycerol, and facilitated the metabolic flux of four modules of the MK-7 synthesis pathway.

**Conclusions:**

In this study, we report for the first time that SinR^quad^ has significant effects on MK-7 synthesis by forming wrinkly and smooth biofilms, upregulating the expression level of most NADH dehydrogenases, and providing higher membrane potential to stimulate the accumulation of the components in the electron transport system.

**Supplementary Information:**

The online version contains supplementary material available at 10.1186/s12934-021-01603-5.

## Introduction

As a highly valuable vitamin K_2_, menaquinone-7 (MK-7) is a polyene compound consisting of a 2-methyl-1,4-naphthoquinone ring structure with a side chain of seven isoprene units [1–3]. It was reported that MK-7 is a component of microbial plasma membranes and plays an important role in electron transport and oxidative phosphorylation [4, 5]. Owing to its good bioavailability, MK-7 functions in protecting human health [6] by preventing osteoporosis [7], arterial calcification, cardiovascular disease, and Parkinson disease [8, 9]. The cost of treating osteoporosis and cardiovascular diseases is high and will likely increase with the aging of populations. The global osteoporosis drug market in 2009 was estimated to be $ 8–9.4 billion, and the market for cardiovascular drugs was estimated to be $ 110–140 billion [1]. Thus, there is a large market for MK and the biosynthesis of MK-7 has received much attention from academia and industry.

In the past decades, many studies have focused on improving the production of MK-7 by microbial fermentation [10–12]. For example, medium components, culture conditions, additives, screening technologies, and the overexpression of exogenous genes in metabolic pathways [13–15] were studied to promote the production of MK. However, these common static metabolic engineering approaches tend to interrupt the elementary metabolic network, resulting in reduced substrate conversion, the accumulation of toxic substances, or reduced product yield [16], the maximum yield of MK-7 was only 35.5 mg/L [17]. Hence, compared with static regulation, dynamic regulation is a promising method achieving a balance between product synthesis and cell growth [18]. Engineered dynamic regulation systems with their capability of adapting to complicated extracellular or intracellular environments are valuable for fine-tuning metabolic flux [19, 20].

In previous studies, dynamic regulation systems have been classified into three categories: biosensors, metabolites response promoters, and quorum-sensing (QS) systems [21]. Biosensors derived from transcription factors bind to DNA to regulate gene expression when interacting with metabolites [22–24]. For example, Xu et al. constructed the malonyl-CoA-responsive biosensors, which could dynamically compensate for the key enzymes in *Escherichia coli* (*E. coli*) to achieve a metabolic balance between cell growth and product formation. They ultimately improved the fatty acid titer 2.1-fold [22]. Metabolite responsive promoters are commonly recognized by screening local promoters that respond to a particular metabolite and regulate gene expression. For instance, Dahl et al. regulated farnesyl pyrophosphate production in the isoprenoid biosynthetic pathway by applying the farnesyl pyrophosphate-responsive promoter. The production of amorphadiene in *E. coli* was improved twofold [25]. The potency of the target product can be greatly increased by using biosensors and metabolic reaction promoters. However, these promoters exist in pathway-specific regulatory systems, severely limiting their widespread applications in many other metabolic pathways [26]. The quorum sensing (QS) system can regulate gene expression according to changes in cell density and overcome the disadvantages of transcriptional factor-based biosensors and metabolite-responsive promoters [27]. The QS system is not dependent on inducers, interventions, or metabolic pathways but rewires the control processes to depend on cell density [28]. For instance, to avoid the toxicity of heterogeneous pathways on cells, an Esa QS circuit with activation and inhibition functions was put forward to produce metabolites without inducers. With the activation of the QS system and dynamic regulation of the biosynthetic pathway by Esa-PesaR, the titer of 4-hydroxyphenylacetic acid increased by 46.4% compared with the static control pathway in *E. coli* [29]. This work proved that QS systems are helpful for fine-tuning metabolic networks.

*B. subtilis* has two QS systems that employ two different processed peptide autoinducers—ComX and competence stimulating peptide (CSP)—to regulate competence and sporulation processes [30]. To explore whether the QS system could promote MK-7 production and how the QS system regulates MK-7 synthesis, a regulator in the QS system which that plays significant role in biofilm formation and MK production simultaneously was selected and its site-directed mutants were constructed. To decipher the mechanism of how this mutant regulates MK-7 synthesis and find additional potential regulators that enhance MK-7 synthesis, the transcriptome was analyzed for expression changes in the QS system, biofilm formation, and MK-7 synthesis pathway of the different mutants. All these results demonstrate that a more wrinkly and smoother biofilm facilitated the flow of glycerol through the biofilm and enhanced MK-7 synthesis. In addition, the electrical hyperpolarization stimulated the synthesis of the electron transport chain components, such as cytochrome c and MK, to ensure the efficiency of electron transfer. This study also proposed some potential regulators of MK-7 biosynthesis, which could provide more ideas for the further enhancement of MK-7 production and deepen our understanding of the molecular mechanism of MK biosynthesis.

## Materials and methods

### Gene knockout, site-directed mutagenesis and growth conditions

**Fig. 1:**
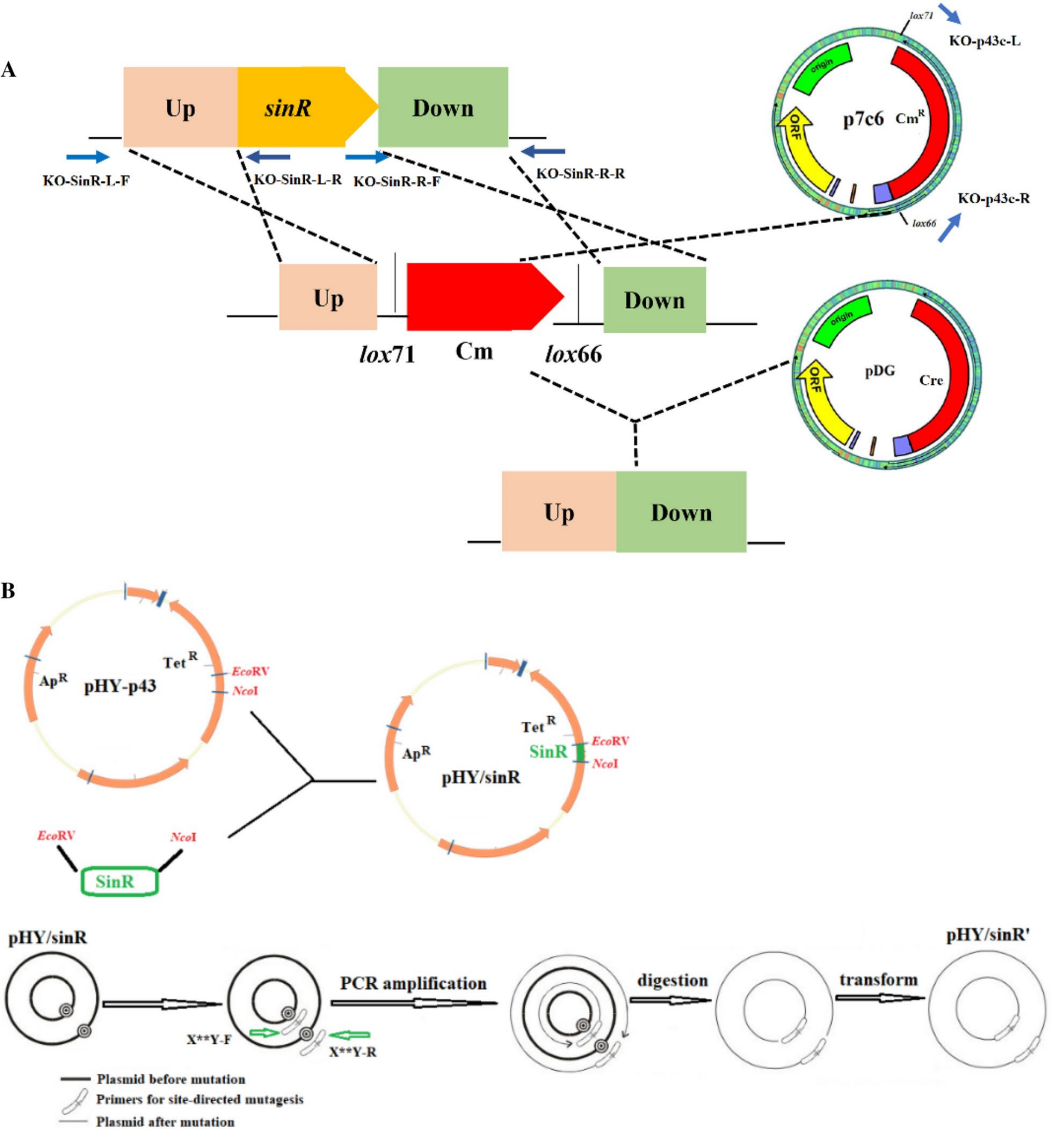
The schematic of construction of KO-SinR (**A**) and E97K, Y101L, W104K, R105S, SinR^quad^ (**B**). X **Y denote E97K, Y101L, W104K, R105S or SinR^quad^

The method used for gene knockout and overexpression was similar to that used by [31]. Briefly, the upstream and downstream sequences (both ~ 1000 bp) flanking the deletion targets were amplified from *B. subtilis*. These two fragments and the lox71-zeo-lox66 cassettes, which were amplified from the plasmid p7C6, were joined by triple-fusion PCR (Fig. 1A). Purified PCR products were used to transform competent *B. subtilis* cells and obtained *sinR* knock out strain (KO-SinR). The primers are shown in Additional file 1: Table S1.

The method used for site-directed mutagenesis was similar to that used by [32]. The PCR reaction was conducted using the PrimeSTAR HS DNA polymerase (Takara, Japan) and the pHY-P43/SinR plasmid as the template DNA. The strains and primers are shown in Additional file 1: Table S1. The PCR product was digested by DpnI (Takara, Japan) at 37 °C for 1 h. The PCR product was transformed into competent cells of *E. coli* DH5α. After the sequence verified, the extracted plasmid was transformed into BS168 KO-SinR cells. E97K, Y101L, W104K, R105S, and SinR^quad^ (Fig. 1B) were obtained for biofilm formation and MK synthesis.

All strains were cultivated in lysogeny broth (LB) liquid culture or on LB agar plates at 37 °C for genetic experiments. The fermentation medium consisted of 5% (w/v) glycerol, 5% (w/v) yeast extract, 5% (w/v) soy peptone, 0.38% (w/v) K_2_HPO_4_, and 0.16% (w/v) KH_2_PO_4_. Ampicillin (100 g/mL) was added to the medium. All chemicals were purchased from Sangon Biotech Co., Ltd (Shanghai, China). MK-7 standard was purchased from ChromaDex (Irvine, CA, USA). Soy peptone, glycerol, and yeast extract were purchased from Sinopharm Chemical Reagent Co., Ltd (Shanghai, China). Methanol, dichloromethane, 2-propanol, and n-hexane were obtained from Sigma-Aldrich (St. Louis, MO, USA).

### Determination of biofilm biomass and MK-7 yield

Overnight cultures were diluted 1:100 into fermentation medium in a 24-well microtiter plate. After incubation at 37 °C for 24 h under microaerobic conditions, the planktonic cells were removed, and the biofilms were washed once with distilled water and then stained by the addition of 0.4% crystal violet (CV) for 30 min at room temperature. CV was then removed with a pipette and biofilms were washed twice with distilled water, solubilized in 30% acetic acid and measured at OD_570nm_ as described previously [33]. Three replicates were examined, and the data were analyzed by the Student’s *t*-test. The MK-7 detection method was the same as in our previous research [34].

### Determination of membrane potential

Membrane potential was determined as described previously [35]. Briefly, BS168 and SinR^quad^ were incubated at 37 °C for 6 days. After cultivation, the cultures were cooled on ice for 10 min, and the cells were harvested by centrifugation (10 min, 8000 rpm, 4 °C). Competent cells were prepared and bacterial suspension was centrifuged (10 min, 8000 rpm, 4 °C). The cells were resuspended in 1 mL of ice-cold buffer solution [distilled deionized water (DDW), 0.9% NaCl or 1 mM MgCl_2_] and 30 mM DiOC_2_(3) was added. After being mixed vigorously by vortex, both mixtures were incubated in the dark for 4 min at room temperature. Finally, the cells were centrifuged (10 min, 8000 g, 4 °C) and washed twice with ice-cold DDW and analyzed by flow cytometry.

### Detection of NADH and NAD^+^ [36]

NADH/NAD^+^ levels of *B. subtilis* were measured using a commercially available kit (MAK037, Sigma-Aldrich, USA) according to the manufacturer’s instructions. NAD^+^ total (NAD^+^ and NADH) or NADH levels were quantified by a colorimetric assay at 450 nm using a SpectraMax i3x (Molecular Devices, USA). The NADH/NAD^+^ ratio was calculated using Eq. (1):

1 $$ {\text{ratio}}\, = \,{\text{C}}_{{{\text{NADH}}}} /\left( {{\text{C}}_{{{\text{total}}}} - {\text{C}}_{{{\text{NADH}}}} } \right) $$

### RNA isolation, library construction, and sequencing

The fermentation broth of BS168 and SinR^quad^ was collected on day 6. The samples were immediately centrifuged for 10 min at 5000 rpm, and then stored at − 80 °C. RNA was extracted by the RNAlock reagent. Total RNA was collected by TRIzol reagent (Invitrogen, USA) and then treated with RNase-free DNase set (NEB, Ipswich, MA, CA, USA). The extracted RNA was detected with an Agilent Bioanalyzer 2100 (Agilent Technologies, Palo Alto, CA, USA). After adding Ribo-Zero Reaction Buffer and Ribo-Zero rRNA Removal Solution (Gram-positive bacteria), the volume was fixed to 40 μL and the reaction was carried out at 68 °C for 10 min. The sample was then placed at room temperature for 5 min. The processed RNA was added to the pre-washed magnetic beads, mixed thoroughly, incubated at room temperature for 5 min and then 50 °C for 5 min, and then immediately placed on a magnetic stand for more than 1 min. The supernatant was removed, and 180 μL water was added to the pellet. Then, 3 M sodium acetate, glycogen (10 mg/mL), and absolute ethanol (600 μL) were added to the solution, which was then placed at – 20 °C for more than 1 h. The solution was centrifuged to obtain a precipitate, which was then dissolved in water to form rRNA-depleted RNA. The RNA library construction from samples at 6 h was completed by the Shanghai Human Genome Research Center. A 2 × 150 bp paired-end sequencing was performed using HiSeq 2000 platform (Illumina, CA, USA) [37].

### RNA-seq data analyses

Clean data was obtained by filtering the original data through SeqPrep/Sickle software to remove the adapter reads, and low-quality and short fragments. Then, clean data were mapped to reference sequences through the Bowtie software based on the Burrows-Wheeler method [38]. The RPKM method was used to estimate the expression levels of gene and transcript by RSEM software [39]. Differentially expressed genes (DEGs) between BS168 and SinR^quad^ were identified by a mathematical statistical model using the edgeR software [40]. The edgeR software can automatically identify and reduce the impact of library construction and sequencing to control the false discovery rate (FDR) [41]. The DEGs were judged through the log2 ratio ≥ 1 and FDR of ≤ 0.05. Then the DEGs were mapped to terms in the gene ontology (GO) and KEGG databases for analyzing the functions and pathways. This analysis of GO functional enrichment used Goatools software with the Fisher precise test method [42]. GO terms with a corrected *p-*value less than 0.05 were regarded as GO terms with significant enrichment in DEGs. In addition, KOBAS software was used for the enrichment analysis of the KEGG pathways [43]. The KEGG pathways with a corrected *p-*value ≤ 0.05 were significantly enriched in DEGs.

## Results

### Site-directed mutagenesis of the transcriptional regulator *sinR* decreases biofilm formation and increases MK-7 synthesis in BS168

**Fig. 2:**
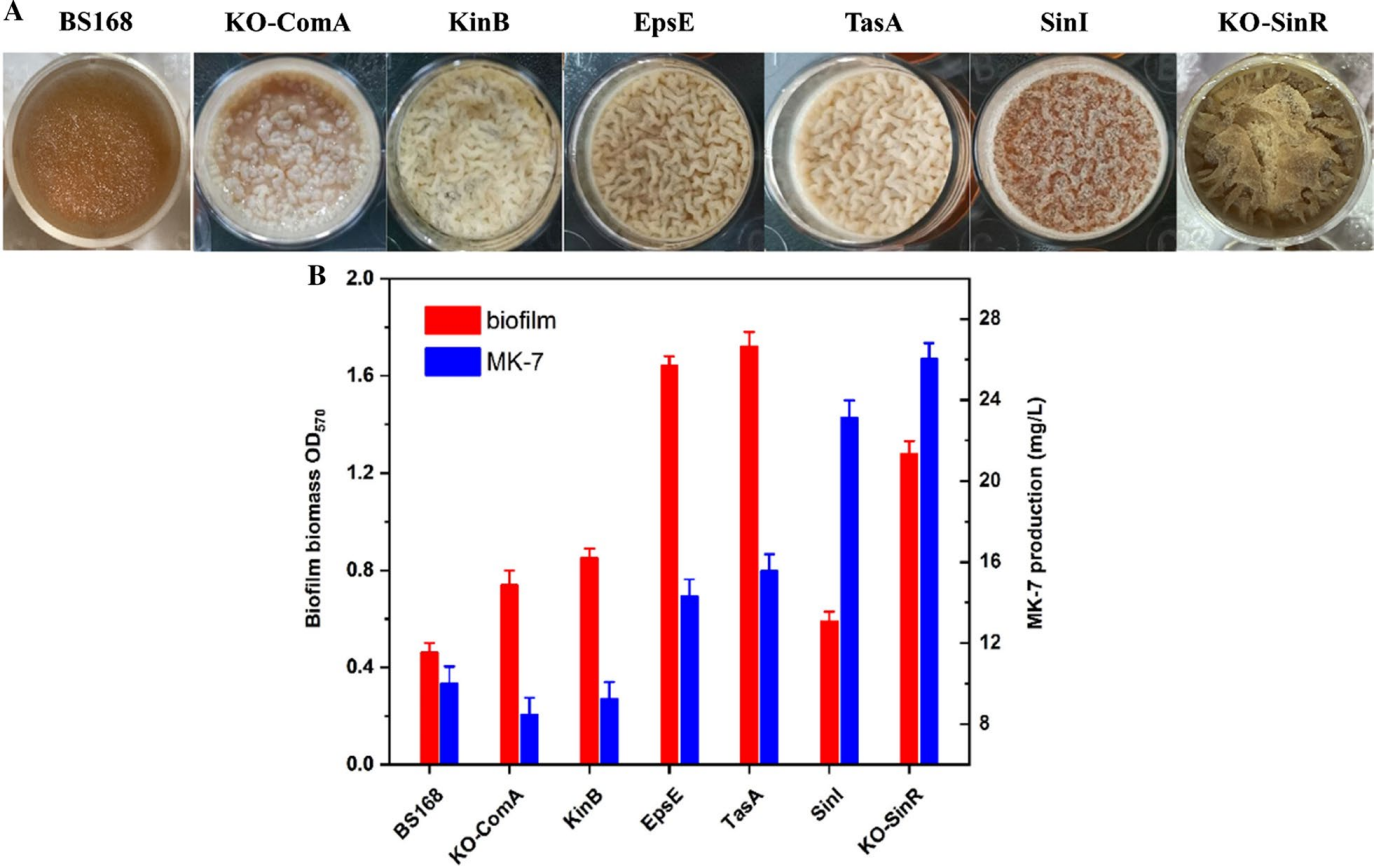
The morphological changes of the biofilm (**A**), the biofilm biomass (**B**) and MK-7 production (**C**) of *comA*, *sinR* knock out strains, and *kinB*, *epsE*, *tasA*, *sinI* overexpression strains

Previous studies found that biofilm formation was beneficial for synthesizing MK-7 in *B. subtilis* [4, 44]. Therefore, we were interested in examining which genes play an important role in affecting both biofilm formation and MK synthesis. A comprehensive mutant library of *B. subtilis* was generated, and the biofilm formation ability and MK-7 synthesis of all mutants were tested (Fig. 2). Our initial screening indicated that deletion of the *sinR* gene resulted in an increased biofilm phenotype in which the biofilm biomass increased 2.8-fold compared to the wild-type (Fig. 2A, B). Furthermore, the concentration of MK-7 increased 2.6-fold after 6 days of cultivation (Fig. 2C). Therefore, the *sinR* gene was chosen for further study.

Some researchers have found that biofilm formation is beneficial for MK-7 synthesis [44], while others said that biofilm formation increased the viscosity, reduced the mass transfer, and subsequently decreased the production of MK-7 [45]. To explore whether there could be a better way to improve MK-7 production by affecting biofilm formation, we used site-directed mutation to alter the ability of SinR to regulate the extracellular matrix.

**Fig. 3:**
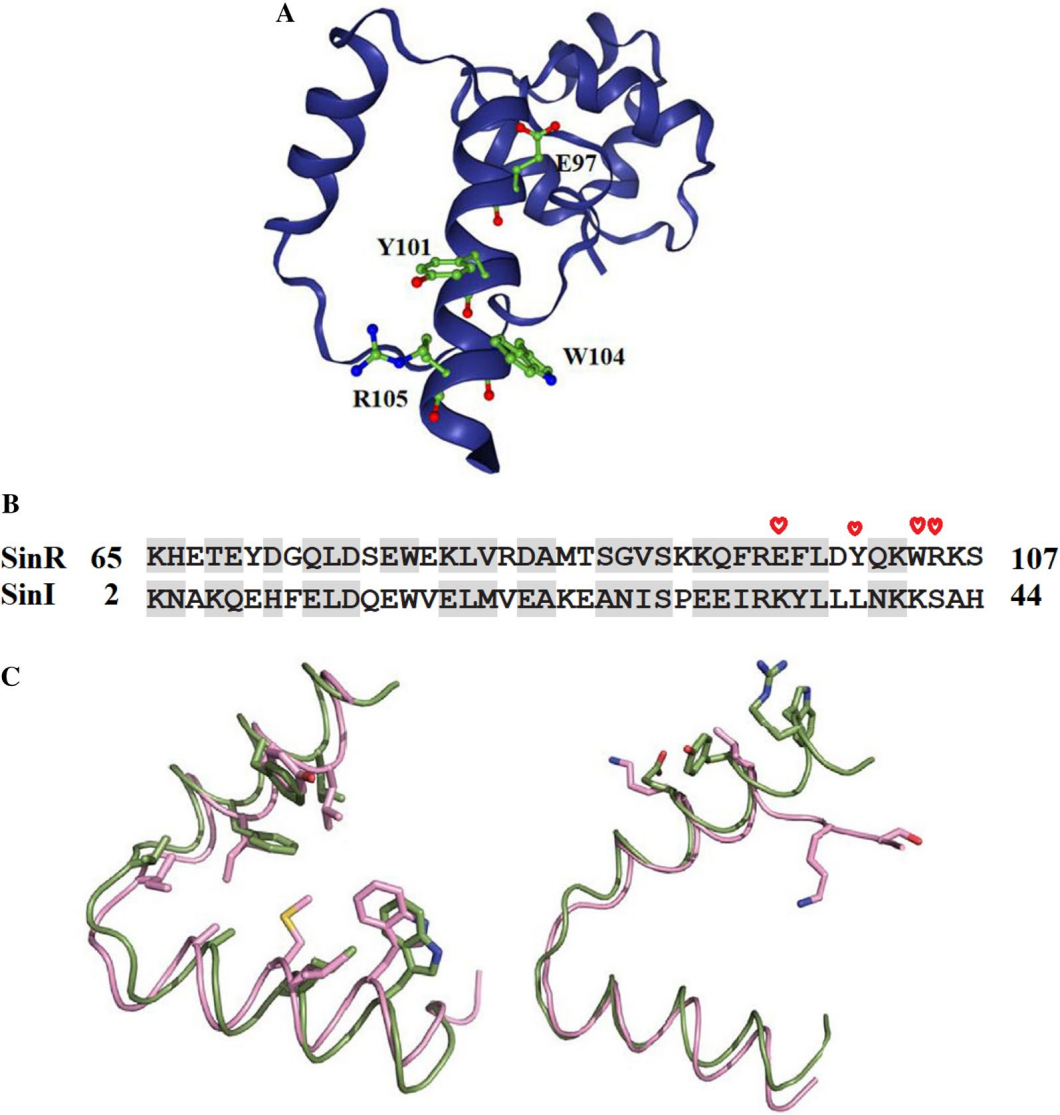
Structure of SinR and the residues of E97, Y101, W104 and R105 (**A**), sequence alignment of the helical hook regions of SinR and SinI (**B**), Structural alignment of SinR (green) and SinI (pink) helical hooks (**C**). Residues are colored based on sequence similarity from Clustal Omega. Stars denote the residues involved in formation of the SinR tetramer interface. These residues in SinR were mutated to the corresponding residue of SinI to generate the SinR^quad^ mutant. Highly conserved amino acids are shown in grey shadow depending on similarity. **C** Left, conserved residues line the dimerization interface. Right, SinR residues necessary for tetramer formation are significantly different in SinI

**Fig. 4:**
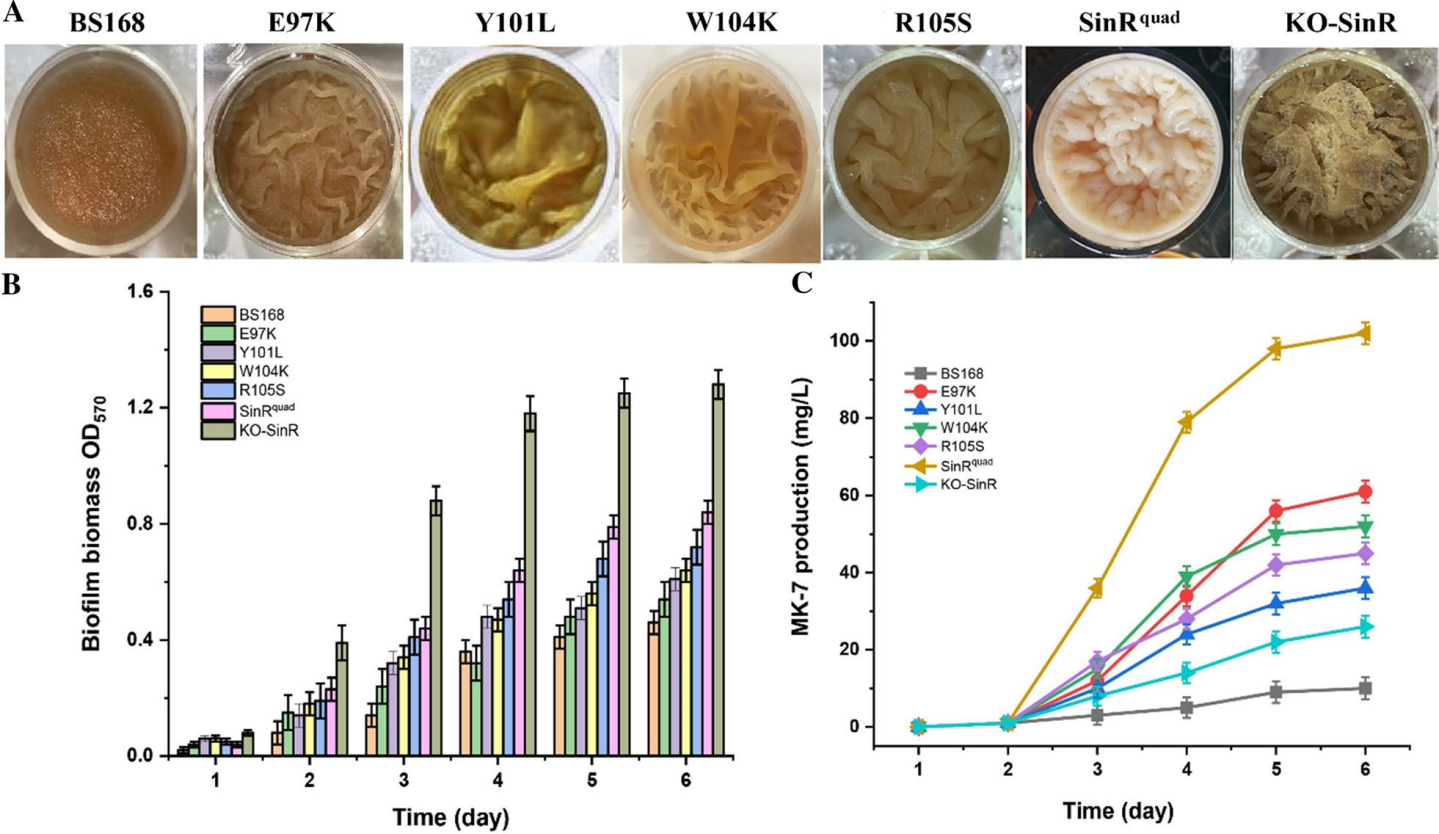
The morphological changes of the biofilm (**A**), the biofilm biomass (**B**) and MK-7 production (**C**) of BS168, E97K, Y101L, W104K, R105S, SinR^quad^ and KO-SinR. All experiments were independently carried out at least three times, and the results were expressed as mean ± standard deviation (SD)

Amino acid residues Glu97, Tyr101, Trp104, and Arg105 in SinR (Fig. 3A) were replaced with Lys34, Leu38, Lys41, and Ser42 in SinI (Fig. 3B) to examine the effects on *B. subtilis*. Figure 4 shows the different biofilm morphologies, biofilm biomasses, and MK-7 production of seven different strains (BS168, E97K, Y101L, W104K, R105S, SinR^quad^, and KO-SinR) after 6 days of incubation. The *B. subtilis* 168 wild-type strain (BS168) made the biofilm look smooth with a few wrinkles. The OD_570nm_ value and the MK-7 production were 0.46 ± 0.04 and 10 ± 0.84 mg/L, respectively. In contrast, a visibly rough and dry biofilm was found in the KO-SinR strain. The OD_570nm_ value and the MK-7 production were 1.28 ± 0.05 and 26 ± 0.78 mg/L, respectively. In addition, E97K, Y101L, W104K, and R105S formed more wrinkles than BS168 but fewer wrinkles than KO-SinR. The OD_570nm_ value of E97K, Y101L, W104K, and R105S increased slightly compared to BS168 but was lower than KO-SinR. However, MK-7 production in four mutants, especially E97K, increased obviously (61.02 ± 0.84 mg/L), which was 2.35-fold that of KO-SinR. When the four sites were mutated simultaneously, we obtained the SinR^quad^ strain. Compared to KO-SinR, SinR^quad^ formed a more wrinkly but smoother biofilm. Although the highest biofilm biomass was obtained by KO-SinR, the maximum MK-7 value (102.56 ± 2.84 mg/L) was obtained by SinR^quad^, which was ten times that of BS168, indicating that the increase in MK-7 production was not induced by biomass growth.

### Illumina HiSeq mRNA sequencing and functional classification of unigenes

**Fig. 5:**
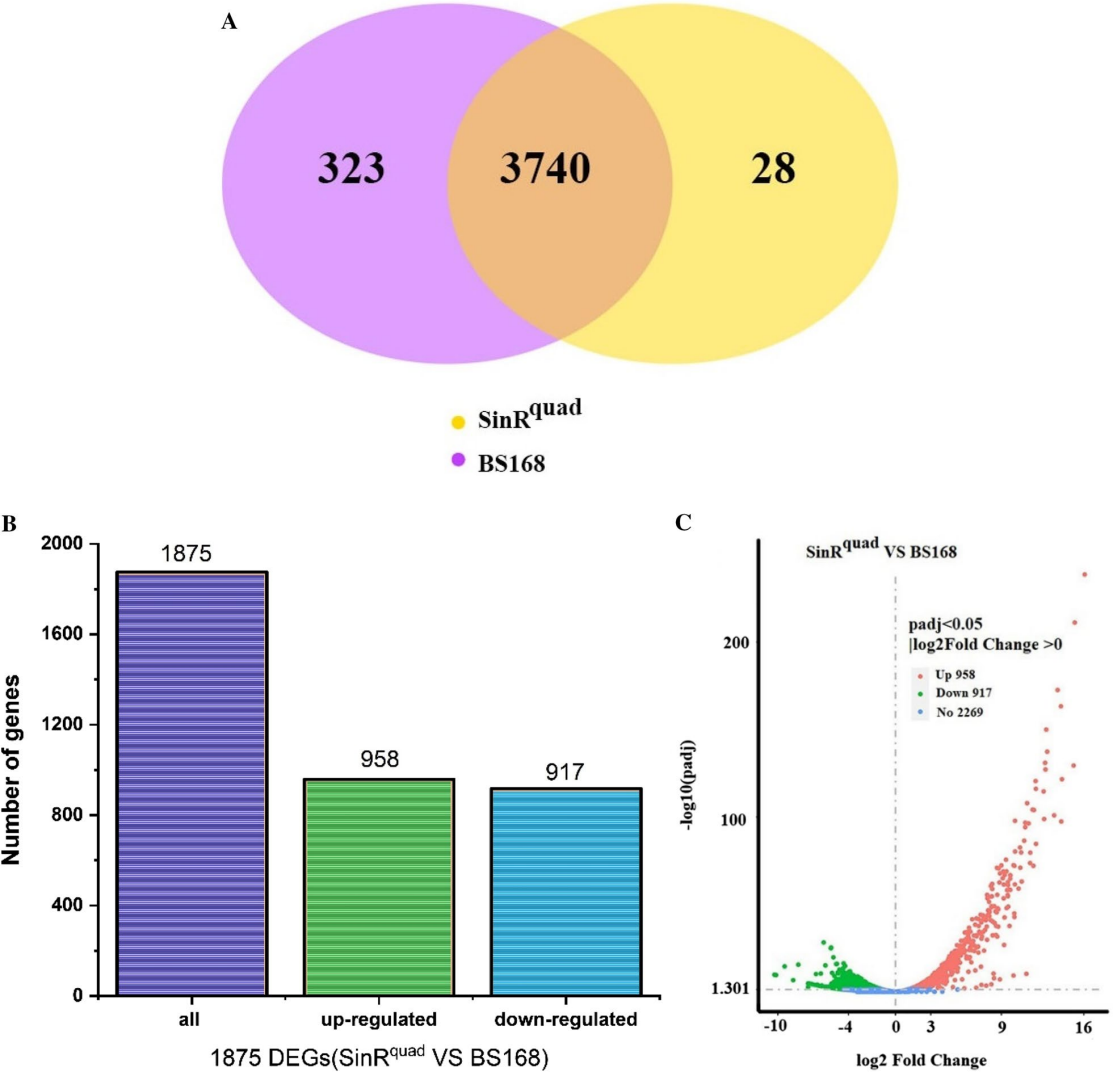
Summary of draft reads of samples by Illumina deep sequencing. **A** Global comparison of BS168 and SinR^quad^ samples by Venn diagrams. **B** Number of up-regulated and down-regulated DEGs of the BS168 and SinR^quad^ samples. **C** Visualization of differential genes (Volcano-plots)

**Fig. 6:**
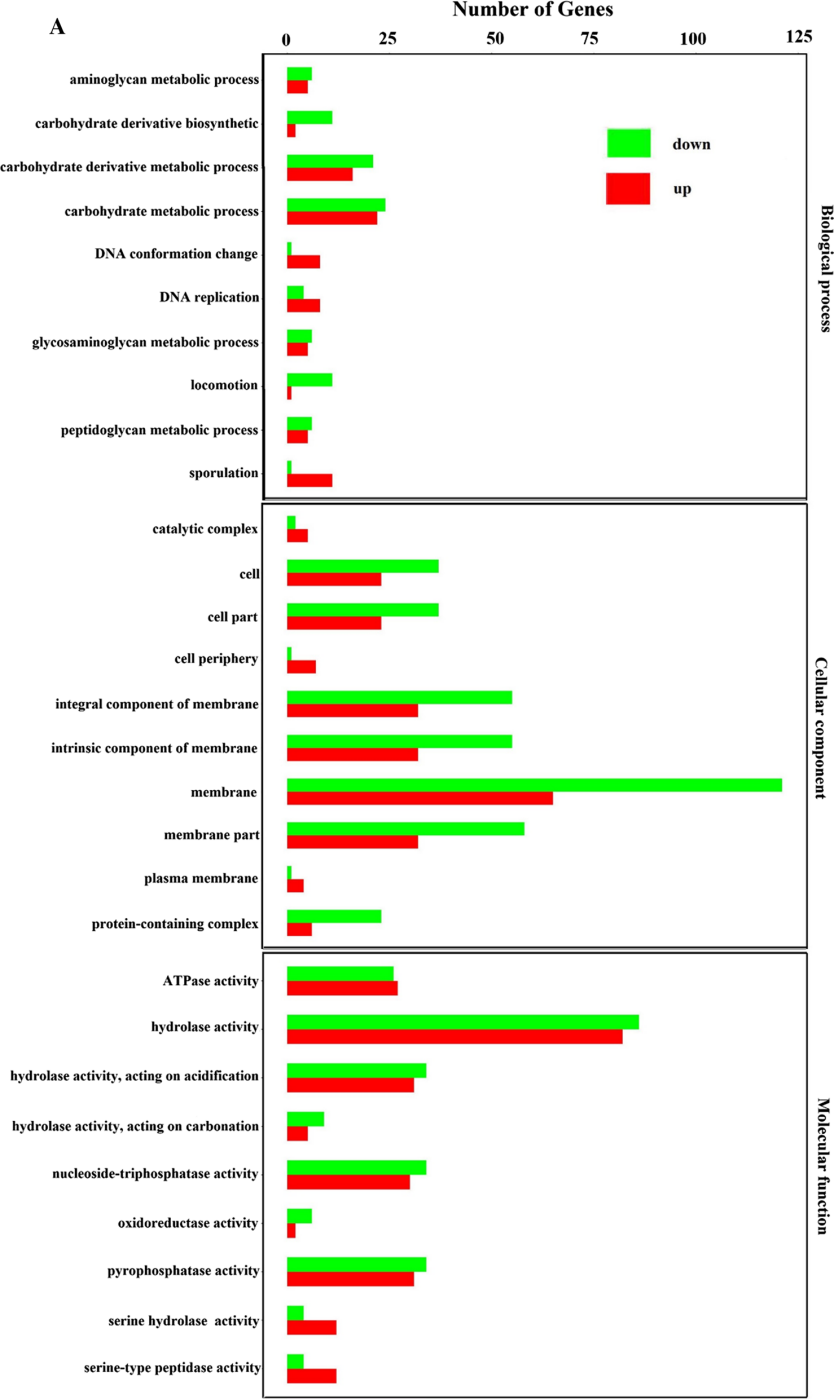

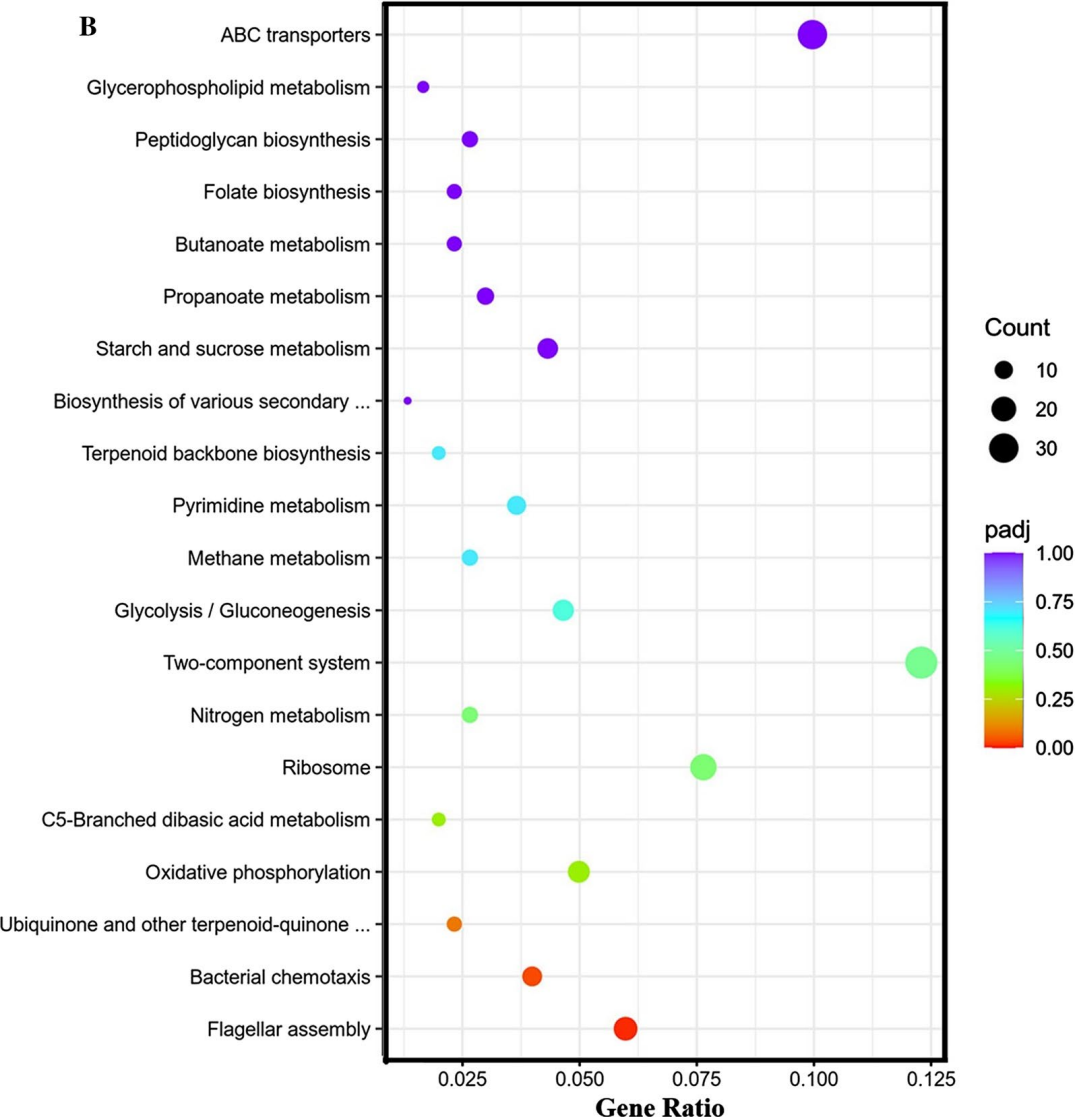
Transcriptomics analysis of differences between BS168 and SinR^quad^. **A** Gene Ontology (GO) functional analysis of differential genes. Unique sequences were assigned to three categories: molecular functional, cellular components and biological process. **B** Kyoto Encyclopedia of Genes and Genomes (KEGG) pathway analysis of functions of differential genes. All experiments were independently carried out at least three times, and the results were expressed as mean ± standard deviation (SD)

In order to explore how SinR^quad^ affects biofilm formation and MK-7 production in *B. subtilis*, the Illumina RNAseq method was used to link genes in related metabolic pathways affected by site-directed mutagenesis of the transcriptional regulator *sinR.* The results showed significant changes in expression levels in 1875 of the 3740 identical genes (Fig. 5B, C). Of these, 958 genes were upregulated, and 917 genes were downregulated (Fig. 5C, D). The functional differences were mainly in “membrane”, “carbohydrate metabolic process”, and “sporulation” (Fig. 6A). The 20 different enrichment pathways included ABC transporters, flagellar assembly, bacterial chemotaxis, ubiquinone and other terpenoid-quinone biosynthesis, and oxidative phosphorylation (Fig. 6B). Because the pathway of the ABC transporters and phosphotransferase system are associated with the state of the cell membrane [46], these differences indicate changes in the external morphology of the colony, menaquinone biosynthesis, respiratory system, and state of the cell membrane in response to site-directed mutagenesis of *sinR.*

### Differential gene expression related to the QS system and biofilm

**Fig. 7:**
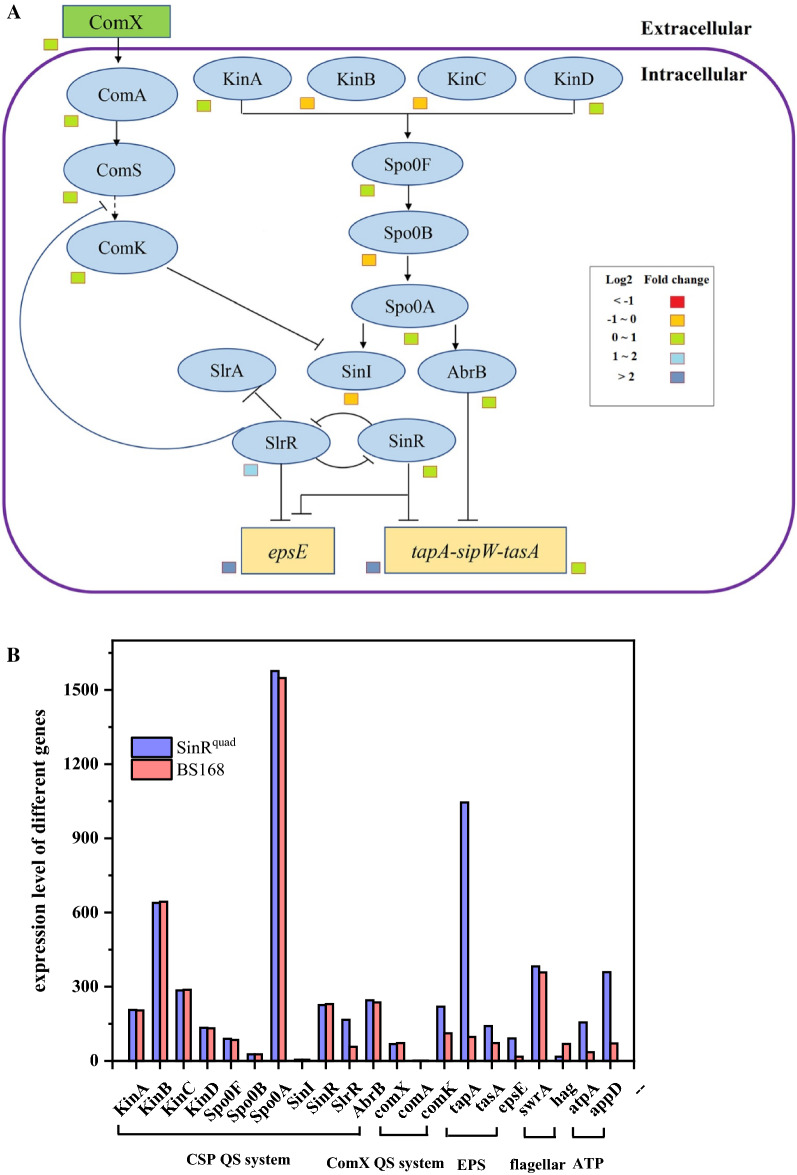
Changes in transcript abundance of genes involved in quorum sensing (QS) system and biofilm between BS168 and SinR^quad^ samples. **A** Transcriptional regulation of the biofilm. **B** The expression level of biofilm forming related genes of SinR^quad^ compared with BS168

To explore why site-directed mutagenesis of *sinR* caused changes in the external morphology of the biofilm, the expression of the QS system and biofilm-related genes of the BS168 and SinR^quad^ were studied. *B. subtilis* uses ComX and CSP QS system to regulate the competence and sporulation processes. It can be seen from Fig. 7B, with regard to the CSP QS system of SinR^quad^, that the expression levels of *spo0A*, *abrB*, *sinI*, and *sinR* were unchanged. The expression of *slrR* was upregulated 1.93-fold. The expressions of *tapA*, *tasA*, and *epsE* were upregulated 9.79-, 0.95-, 4.42-fold, respectively. The upregulation of *tapA*, *tasA*, and *epsE* implied that the extracellular matrix, which constituted the biofilm, was promoted in SinR^quad^.

With regard to the ComX QS system, the expressions of *comX*, *comA*, and *comS* were unchanged, indicating that expression of the ComX QS system and ComX regulated the biofilm genes and were unaffected by site-directed mutagenesis of SinR*.*

Numerous previous studies have reported that SinR is a positive effector of motility and cell separation, which is not conducive to the formation of biofilms [47]. However, it was still unknown whether the expression of flagella and chemotaxis proteins would change with site-directed mutagenesis of SinR. Therefore, the expression levels of the *swrA* and *hag* genes were investigated. As we know, *swrA* encodes proteins for the hook-basal body of flagella, chemotaxis, and the flagellum-associated sigma factor σ^D^ [48]. The *hag* gene, a late-flagellar gene, encodes the flagellar filament structural protein flagellin, which is responsible for swimming motility [49]. Figure 7B shows that the relative transcription of the *swrA* gene had no change between SinR^quad^ and BS168. However, expression of the *hag* gene was downregulated by 75.65%. Therefore, site-directed mutagenesis of E97K, Y101L, W104K, and R105S of *sinR* hindered the expression of the late-flagellar gene.

The *appD* gene, which encodes an oligopeptide ABC transporter (ATP-binding protein) [50], was upregulated 4.07-fold, implying that membrane transport was promoted in SinR^quad^. Therefore, SinR^quad^ promoted the synthesis of extracellular polymeric substances through the CSP QS system, improved membrane transport, inhibited the swimming motility of late-flagellar, and, thus, promoted the biofilm biomass.

### Differential gene expression related to MK-7 biosynthesis

**Fig. 8:**
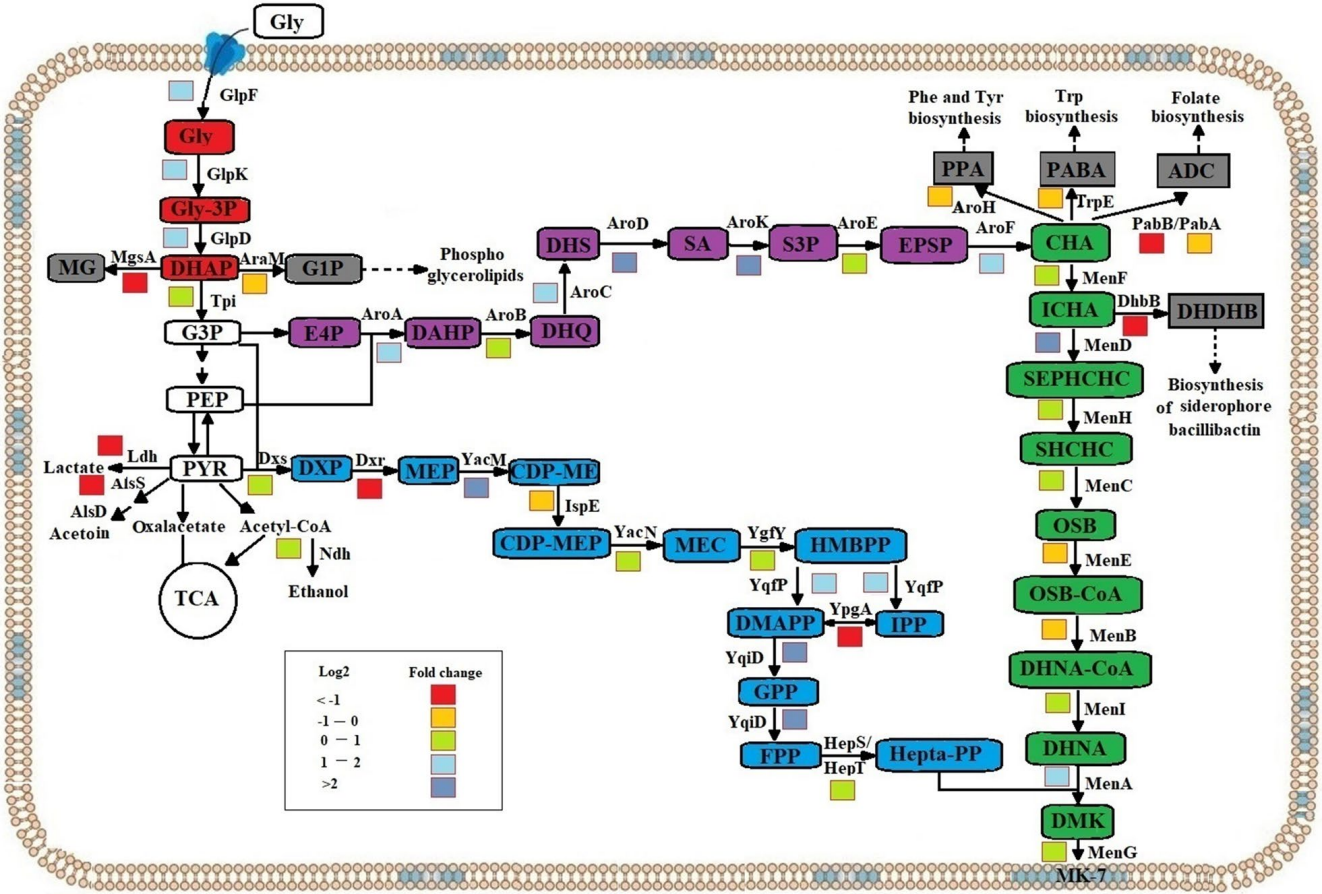
Changes in transcript abundance of genes involved in MK-7 metabolic pathway. The biosynthesis pathway from glycerol to MK-7 in *B. subtilis* 168 was categorized into four modules I–IV. Enzymes: Module I (glycerol metabolism pathway, marked in red): *GlpF* glycerol uptake facilitator, *GlpK* glycerol kinase, *GlpD* glycerol-3-phosphate dehydrogenase, *Tpi* triosephosphate isomerase. Module II (MEP pathway, marked in blue): *Dxs* 1-deoxyxylulose-5-phosphate synthase, *Dxr* 1-deoxyxylulose-5-phosphate reductoisomerase, *YacM* 2-C-methylerythritol 4-phosphate cytidylyltransferase, *IspE* 4-diphosphocytidyl-2-C-methylerythritol kinase, *YacN* 2-C-methylerythritol 2,4-cyclodiphosphate synthase, *YgfY* 4-hydroxy-3-methylbut-2-enyl diphosphate synthase, *YqfP* 4-hydroxy-3-methylbut-2-enyl diphosphate reductase, *YpgA* isopentenyl-diphosphate δ-isomerase, *YqiD* farnesyl diphosphate synthase. Module III (SA pathway, marked in purple): *AroA* 3-deoxy-7-phosphoheptulonate synthase, *AroB* 3-dehydroquinate synthase, *AroC* 3-dehydroquinate dehydratase, *AroD* shikimate dehydrogenase, *AroK* shikimate kinase, *AroE* 3-phosphoshikimate 1-carboxyvinyltransferase, *AroF* chorismate synthase. Module IV (MK-7 pathway, marked in green): *MenF* isochorismate synthase, *MenD* 2-succinyl-5-enolpyruvyl-6-hydroxy-3-cyclohexene-1-carboxylate synthase, *MenH* 2-succinyl-6-hydroxy-2,4-cyclohexadiene-1-carboxylate synthase, *MenC* *o*-succinylbenzoate synthase, *MenE* *o*-succinylbenzoic acid-CoA ligase, *MenB* 1,4-dihydroxy-2-naphthoyl-CoA synthase, *MenI* 1,4-dihydroxy-2-naphthoyl-CoA hydrolase, *MenA* 1,4-dihydroxy-2-naphthoate heptaprenyltransferase, *MenG* demethylmenaquinone methyltransferase, *HepS/HepT* heptaprenyl diphosphate synthase component I/II. Other related pathways (branched metabolic pathway, marked in grey): *Ldh* lactate dehydrogenase, *AlsS* acetolactate synthase, *AlsD* acetolactate decarboxylase, *AroH* chorismate mutase, *TrpE* anthranilate synthase, *PabB/PabA* *para*-aminobenzoate synthase component I/II, *DhbB* bifunctional isochorismate lyase/aryl carrier protein. Abbreviations of metabolites: *Gly* glycerol, *Gly-3P* glycerol-3-phosphate, *CDP-ME* 4-(cytidine 5′-diphospho)-2-C-methylerythritol, *CDP-MEP* 2-phospho-4-(cytidine 5′-diphospho)-2-C-methylerythritol, *PPA* prephenate, *PABA* *para*-aminobenzoic acid, *ADC* 4-amino-4-deoxychorismate, *DHDHB* (2S,3S)-2,3-dihydro-2,3-dihydroxybenzoate, *MG* methylglyoxal, *G1P* glycerol-1-phosphate, *G3P* glyceraldehyde-3-phosphate, *PEP* phosphoenolpyruvate, *PYR* pyruvate, *E4P* erythrose 4-phosphate, *DAHP* 3-deoxy-arabino-heptulonate 7-phosphate, *DHQ* 3-dehydroquinate, *DHS* 3-dehydroshikimate, *SA* shikimate, *S3P* shikimate 3-phosphate, *EPSP* 5-*O*-(1-carboxyvinyl)-3-phosphoshikimate, *CHA* chorismate, *DXP* 1-deoxyxylulose-5-phosphate, *MEP* methyl-erythritol-4-diphosphate, *HMBPP* 1-hydroxy-2-methyl-2-butenyl 4-diphosphate, *DMAPP* dimethylallyl diphosphate, *IPP* isopentenyl diphosphate, *GPP* geranyl diphosphate, *FPP* farnesyl diphosphate, *ICHA* isochorismate, *SEPHCHC* 2-succinyl-5-enolpyruvyl-6-hydroxy-3-cyclohexene-1-carboxylate, *SHCHC* 2-succinyl-6-hydroxy-2,4-cyclohexadiene-1-carboxylate, *OSB* 2-succinylbenzoate, *OSB-CoA* 2-succinyl benzoyl-CoA, *DHNA-CoA* 1,4-dihydroxy-2-naphthoyl-CoA, *DHNA* 1,4-dihydroxy-2-naphthoate, *DMK* 2-demethylmenaquinone, *MK-7* menaquinone-7

The biosynthesis pathway of MK-7 in *B. subtilis* presented in Fig. 8 can be categorized into four modules, namely the glycerol metabolism pathway (Module I), the methylerythritol phosphate (MEP) pathway (Module II), the shikimate (SA) pathway (Module III), and the MK-7 pathway (Module IV) [51].

Previous studies comparing the effect of four carbon sources (i.e., soluble starch, sucrose, glucose, and glycerol) on MK-7 synthesis and growth of *B. subtilis natto* found that the presence of glycerol in the media resulted in higher MK-7 production [10, 52]. Therefore, the first step in the biosynthesis pathway was the uptake of glycerol, which is catalyzed in an energy-independent manner by a membrane channel protein, the glycerol facilitator (GlpF) [53]. The main pathway of glycerol dissimilation involves a glycerol kinase (GlpK) that phosphorylates glycerol to glycerol-3-phosphate (Gly-3P), and a Gly-3P dehydrogenase (GlpD) that oxidizes Gly-3P to dihydroxyacetone phosphate (DHAP), an intermediate in glycolysis. In the glycerol metabolism pathway, the expression of *glpF*, *glpk*, and *glpD* were upregulated 1.21-, 2.20-, and 1.11-fold, respectively. In addition, methylglyoxal synthase (MgsA) and glycerol-1-phosphate dehydrogenase (AraM), which catalyze the conversion of DHAP to methylglyoxal (MG) and the reduction of DHAP to glycerol-1-phosphate (G1P), were downregulated by 97% and 46%, respectively. The result indicated that SinR^quad^ could increase the consumption of the substrate glycerol and weaken the other two branch pathways of DHAP, allowing DHAP to flow into glycolysis as much as possible.

Module II provides isopentenyl diphosphate (IPP) and its isomer dimethylallyl diphosphate (DMAPP) for the biosynthesis of isoprenoids, which is the precursor of the side chain of MK-7. However, studies on the key enzymes in the MEP pathway in *B. subtilis* are rare. Figure 8 shows that most of the enzymes in Module II were upregulated in the SinR^quad^ strain. As the first rate-limiting enzyme in the MEP pathway, 1-deoxy-d-xylulose 5-phosphate reductoisomerase (Dxs), which catalyzes the reaction of pyruvate and glyceraldehyde 3-phosphate to form 1-deoxy-d-xylose-5-phosphate (DXP), was upregulated 0.91-fold. The result was consistent with a previous study that found overexpressing *dxs* could increase the yield of isoprene by 40% over that of the wild-type *B. subtilis* [54]. In addition, Yqfp, which catalyzes the synthesis of isopentenyl diphosphate (IPP) and its isomer dimethylallyl diphosphate (DMAPP), was upregulated 1.95-fold. Especially, YacM and Yqid, which catalyze the reaction of MEP and cytosine triphosphate (CTP) to form 4-diphosphocytidyl-2-C-methyl-d-erythritol (CDP-ME), and polymerize IPP to farnesyl pyrophosphate (FPP), were upregulated 2.26-fold and 2.06-fold, respectively.

Module III provided chorismate (CHA), the precursor of the main chain of MK-7. CHA is essential in cellular metabolism for providing the precursors for the biosynthesis of three aromatic amino acids: tyrosine (Tyr), phenylalanine (Phe), and tryptophan (Trp) [55]. The four enzymes AroA, B, C, and D can catalyze D-erythrose 4-phosphate and phosphoenolpyruvate to form shikimate. Shikimate will be converted to chorismate by AroK, E, and F. Figure 7 shows that *aroA, B, C, D, K, E,* and *F* were upregulated 1.47-, 0.75-, 1.64-, 3.77-, 8.30-, 1.08-, and 0.14-fold, respectively. The genes encoding AroH, TrpE, PabA, and PabB, which are involved in the synthesis of three aromatic amino acids, were downregulated by 41%, 29%, 21%, and 62%, respectively. The result indicated that AroA, D, and K played an important role in CHA synthesis. This was consistent with a previous study showing that simultaneous overexpression of *aroA* and *aroK* in *B. subtilis* resulted in a twofold increase in MK-7 compared with strain BS168 [56].

Module IV is the last pathway and is for MK-7 synthesis. Seven enzymes (MenF, D, H, C, E, B, I) for the synthesis of 1,4-dihydroxy-2-naphthoyl-CoA (DHNA-CoA) in the MK-7 pathway were detected with different expression changes (Additional file 2: Table S2). Five genes were upregulated, especially *menD* whose expression level was highly regulated by 3.87-fold. Finally, both *menA* and *menG*—which could combine the isoprene side chain and naphthoquinone ring and then catalyze the methylation to form MK-7—were upregulated 2.03- and 0.72-fold, respectively, in the SinR^quad^ strain. It is consistent with our previous studies where overexpression of *menG/ubiE* in *Elizabethkingia meningoseptica* enhanced the MK content 1.41-fold [34]. These results provide a new idea for further understanding the effect of SinR^quad^ on the biosynthesis of MK-7 at the transcriptional level.

### Differential gene expression related to the cell membrane

**Fig. 9:**
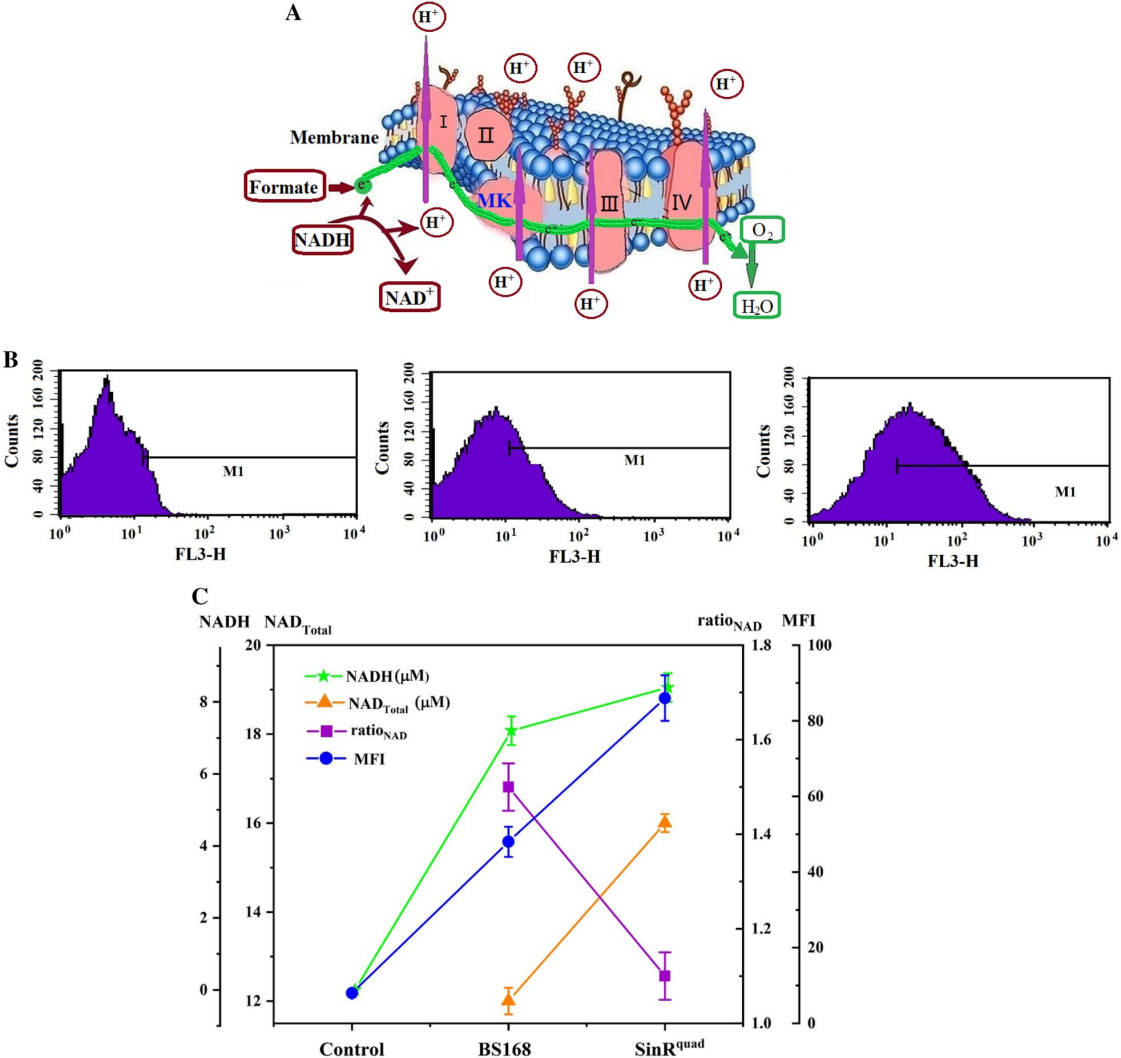

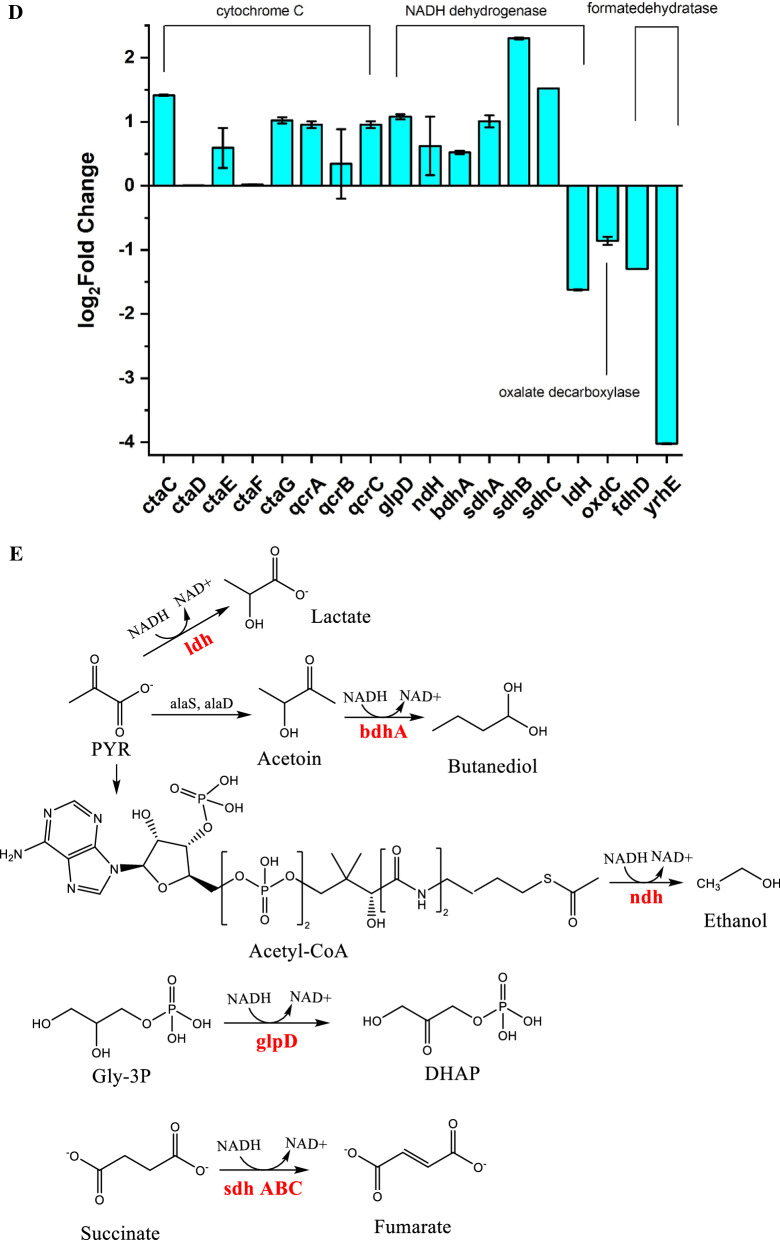
Schematic diagram of electron transfer chain in *B. subtilis*. Electrons are extracted under action of NADH dehydrogenase and formate. MK-7 and cytochrome c act as electron transport carriers, and finally electrons are delivered to oxygen to form water (**A**). Membrane potential evaluated by Flow cytometry of dead cell (Control), BS168 and SinR^quad^ (**B**). Changes in the membrane potential evaluated by mean fluorescence intensity (MFI), total NAD content, NADH content yield and NADH/NAD^+^ ratio in BS168 and SinR^quad^ (**C**). Expression level of NADH dehydrogenase of SinR^quad^, compared with BS168; positive numbers mean upregulation; negative numbers mean downregulation (**D**). Catalytic reaction of NADH reduction in cells, including l-lactate dehydrogenase (*ldH*), (*R*,*R*)-butanediol dehydrogenase (*bdhA*), NADH dehydrogenase (*ndH*), aerobic glycerol-3-phosphate dehydrogenase (*glpD*), and succinate dehydrogenase flavoprotein (*sdhABC*) (**E**). All experiments were independently carried out at least three times, and the results were expressed as mean ± standard deviation (SD)

MK-7 is a component of bacterial cell membranes and plays an important role in electron transfer and respiration. A schematic of electron flow mediated by MK in *B. subtilis* is illustrated in Fig. 9A. Respiration occurs in the cell membrane of Gram-positive bacteria. Electron donors, with the help of an enzyme, transfer two electrons to MK and cytochrome c. MK and cytochrome c, with the help of another enzyme, in turn transfer these two electrons to an electron acceptor oxygen to form water. To determine changes in electron transfer and respiration in the cell membrane of dead cells (Control), BS168, and SinR^quad^, the membrane potential, the expression of genes encoding the cytochromes, and MK-7 production were measured after 6 days of cultivation. We observed that the mean fluorescence intensity (MFI) of SinR^quad^ rose to 86, which was 1.8-fold that of BS168 (Fig. 9B, C). A rise in the magnitude of the membrane potential is referred to as electrical hyperpolarization in SinR^quad^. In addition, the expression levels of the *ctaC-G* operator and *qcrA-C* operator, which encode the cytochromes [57], were upregulated (Fig. 9D), and the Log2 fold changes were all greater than 0. MK-7 production was also ten times that of BS168 (Fig. 4C). These results proved that SinR^quard^ could deliver more electrons and promote respiration in *B. subtilis*. A previous study found that electrons were formed during the conversion of oxalate to formate and CO_2_ [58]. Therefore, we tested the expression level of oxalate-decarboxylase (OxdC), which, with the help of Mn and O_2_, catalyzes the conversion of oxalate to formate and CO_2_. However, the expression level of *oxdC* demonstrated a 45% decrease. In addition, the expression levels of *fdhD* and *yrhE*, which encode formate dehydratase and are used to oxidize formate to CO_2_ and electrons, were downregulated by 60% and 94%, indicating that electrical hyperpolarization of SinR^quard^ was not due to this process. Therefore, we speculate that there may be other processes that can donate large amounts of electrons. It has been reported that NADH is the most important electron donor. It donates electrons under the action of NADH dehydrogenase and transfers electrons to the electron transport system (ETM) and pumps protons out of the cell [36]. The process of producing electrons is shown in Fig. 9E. Consequently, NAD^+^ total (NAD^+^ and NADH) and NADH levels and the NADH/NAD^+^ ratio were quantified by a colorimetric assay. The NAD^+^ total and NADH levels increased 1.33- and 1.17-fold, respectively, while the NADH/NAD^+^ ratio decreased by 27%. This result was consistent with a previous study, which found that the ratio of NADH/NAD^+^ in *B. subtilis* was inversely proportional to the concentration of MK-7 [36]. The expression levels of NADH dehydrogenases (i.e., *ndH*, *bdhA*, *sdhA-C*, *idH*, and *glpD*) were also tested. As shown in Fig. 9D, the expression levels of most NADH dehydrogenases were upregulated. Especially, *sdhA*, *sdhB*, *sdhC*, and *glpD*, increased 1.01-, 3.93-, 1.87-, and 1.11-fold, respectively.

## Discussion

Previous studies have found that biofilm formation is beneficial for MK-7 synthesis in *B. subtilis* [44]. However, others supposed that biofilm formation increases the viscosity, reduces the mass transfer, and subsequently decreases MK-7 production [45]. To explore whether MK-7 synthesis is affected by biofilm formation and whether there is a quantitative correlation between MK-7 synthesis and biofilm formation, we used transcriptomic analysis to target SinR (a QS system transcriptional regulator). We revealed many potential genes related to MK-7 biosynthesis, spore and biofilm formation, and the respiratory oxidation system in *B. subtilis*. As far as we know, this study is the first report to illustrate the relationship between active sites of SinR and MK-7 biosynthesis, biofilm formation, and the respiratory oxidation system in *B. subtilis*. This work also provides new information for the further enhancement of MK-7 production, which is essential for the industrialized application of MK-7.

As we know, SinR is a constitutively expressed transcriptional regulator and has been identified as a master regulator of biofilm formation [46]. DNA binding by SinR leads to repression of the *epsA-O* and *tapA-sipW-tasA* operons by the CSP QS system [46], both of which are involved in the production of biofilm matrix polysaccharides and proteins. Our study confirmed this conclusion since KO-SinR caused an obviously increased biofilm phenotype, and the biofilm biomass increased 2.8-fold compared to the wild-type (Fig. 2A, B).

SinI is an antagonist of SinR by forming SinI-SinR dead-end complex and sequestering free SinR, thereby ensuring biofilm formation. A previous study found that SinI displaces a SinR monomer from its homotetramer complex [59]. Despite the SinR dimer interface and the SinI dimer interface being highly homologous in sequence and structure (Fig. 3B, C), the four key residues involved in SinR tetramer formation (E97, Y101, W104, R105) are significantly different in SinI. Furthermore, Glu97 and Arg105 as well as pi-stacking from Tyr101 and Trp104 were found to form salt bridges to join adjacent helices [60]. Therefore, Glu97, Tyr101, Trp104, and Arg105 in SinR were replaced with Lys34, Leu38, Lys41, and Ser42 in SinI and mimicked the SinR tetramer interface with DNA binding. A previous study found that equal molar ratios of SinR and SinI resulted in a complete disruption in the SinR homotetramer with a broad 1.7 S distribution [60]. Our results verified the prediction that when SinR is replaced by a SinR mutation, the SinR homotetramer may be disrupted, which affects SinR DNA-binding activity and promotes biofilm formation. Five mutants (E97K, Y101L, W104K, R105S, and SinR^quad^), especially SinR^quad^, which contain four point mutations and thus disrupt the SinR homotetramer more severely, formed more wrinkly and smoother biofilms compared to BS168.

Different biofilm morphologies formed by site-directed mutagenesis of SinR affect the bacteria to suit outside environmental changes and nutrient transport. SinR^quad^ formed a more wrinkled biofilm surface compared to BS168 and the other five mutants, while the wrinkles formed a network of interconnected channels with a low resistance to liquid flow. Pressure within the channels was less than the atmospheric pressure outside. This pressure gradient could drive flow through the channels, facilitate nutrient flow through the biofilm [61], and finally affect the synthesis of secondary metabolites. In our study, we found the expressions of *glpF*, *glpk*, and *glpD* in SinR^quad^ were upregulated 1.21-, 2.20-, and 1.11-fold, respectively, indicating that SinR^quad^ formed a more winkled biofilm that facilitated glycerol flow through the biofilm. A previous study found that the glycerol dissociation pathway is important for MK synthesis. When *glpK* and *glpD* were overexpressed, MK-7 titer had a 10% increase [51]. KO-SinR could also form a wrinkled biofilm (Fig. 3A). However, the biofilm surface was drier than the other mutants, which means that motile cells become spores that were not beneficial for metabolite synthesis. This was one reason why MK-7 synthesis by SinR^quad^ and E97K were 10-times and 2.35-times higher than KO-SinR.

Transcriptomic analysis showed that the pathways such as ABC transporters, bacterial chemotaxis, and oxidative phosphorylation changed significantly, indicating that the respiratory system and state of the cell membrane were changed obviously in SinR^quad^. A previous study discovered that changing the state of the membrane in *Escherichia coli* could increase the synthesis of MK-8 [62]. Our study verified this prediction by quantitatively determining the membrane potential and respiration of SinR^quad^ and BS168. MFI of SinR^quad^ rose to 86, which was 1.8-fold that of BS168 (Fig. 9B, C). A rise in the magnitude of the membrane potential is referred to as electrical hyperpolarization in SinR^quad^, which means more protons and electrons were produced in SinR^quad^ compared to BS168. The extra electrons were donated by NADH under the action of NADH dehydrogenase and transferred to the electron transport system (ETM). This was indicated by the 1.33- and 1.17-fold increase in NAD^+^ total and NADH levels, respectively. The expression levels of most NADH dehydrogenases were upregulated. In particular, *sdhA*, *sdhB*, *sdhC*, and *glpD*, increased 1.01-, 3.93-, 1.87-, 1.11-fold, respectively. The increase in electron and electrical hyperpolarization stimulated the synthesis of the electron transport chain components, such as cytochrome c and MK, to ensure electron transfer efficiency [44]. Active ETM was another reason why MK-7 synthesis in SinR^quad^ was ten times higher than in KO-SinR and BS168.

Many researchers have found that MenA and MenD play an important role in MK synthesis in different strains [51, 63]. Our study also found that the expression of *menA* and *menD* increased 2.04 times and 3.87 times in SinR^quad^, respectively, confirming again that the step connecting the isoprene side chain and naphthoquinone ring by MenA, and the catalysis of isochorismate (ICHA) to 2-succinyl-5-enolpyruvyl-6-hydroxy-3-cyclohexene-1-carboxylate (SEPHCHC) by MenD, were the rate-limiting steps. More glyceraldehyde 3-phosphate entering the MEP pathway is beneficial for the formation of the polyprenyl side chain, and entering the SA pathway is conducive to the synthesis of the naphthoquinone ring. According to previous research, the biosynthesis of the polyprenyl side chain, rather than biosynthesis of the naphthoquinone ring (1,4-dihydroxy-2-naphtoic acid), limited total MK production in *Lactococcus lactis* [64]. However, in our study, we found most of the genes in the biosynthesis of the naphthoquinone ring were upregulated and more glyceraldehyde 3-phosphate entered the SA pathway. A possible reason could be that different strains have different metabolic regulation patterns in the MK-7 metabolic pathway. Therefore, the regulatory mechanisms of this pathway need to be further explored.

In summary, we preliminarily determined the mechanism of how site-directed mutagenesis of the transcriptional regulator SinR affects the biosynthesis of menaquinone in *Bacillus subtilis*. We did this by analyzing the QS system, biofilm formation, cell membrane state, and MK-7 synthesis pathway of SinR^quad^ and BS168. E97K, Y101L, W104K, R105S, and SinR^quad^ disrupted the SinR homotetramer, thus forming a more wrinkly and smoother biofilm than BS168. On the one hand, the more wrinkled and smoother biofilm facilitated glycerol flow through the biofilm and enhanced MK-7 synthesis. On the other hand, electrical hyperpolarization stimulated the synthesis of the electron transport chain components, such as cytochrome c and MK, to ensure electron transfer efficiency.

## Supplementary Information

**Additional file 1: Table S1**. Strains and primers used in this work

**Additional file 2: Table S2**. Genes significantly changed in the SinR^quad^ compared to *Bacillus subtilis* 168

## Data Availability

The data generated and analyzed during this study are included in the article and its Additional files 1 and 2.
