# Supplementary material for: Site-directed mutagenesis of the quorum-sensing transcriptional regulator SinR affects the biosynthesis of menaquinone in Bacillus subtilis
Source: Microb Cell Fact. 2021 Jun 7;20:113. doi: 10.1186/s12934-021-01603-5 (PMC8183045; doi:10.1186/s12934-021-01603-5)
Supplement: Supplementary file 1 — Additional file 1: Table S1. Strains and primers used in this work [file 12934_2021_1603_MOESM1_ESM.docx]

**Table S1. Strains and primers used in this work**

| Strain | Properties and genotype | Sources |
| --- | --- | --- |
| *B. subtilis 168* | *trpC2* | Granted by Jiangnan University |
| E97K | BS168-*sinR,* the glutamine at position 97 was changed to a lys residue in *B. subtilis* 168 | This work |
| Y101L | BS168-*sinR,* the Tyr at position 101 was changed to a leu residue in *B. subtilis* 168 | This work |
| W104K | BS168-*sinR,* the Trp at position 104 was changed to a lys residue in *B. subtilis* 168 | This work |
| R105S | BS168-*sinR,* the Arg at position 105 was changed to a ser residue in *B. subtilis* 168 | This work |
| SinR^quad^ | BS168-*sinR,* the glutamine at position 67 was changed to a lys residue, the Tyr at position 101 was changed to a leu residue, the Trp at position 104 was changed to a lys residue, and the Arg at position 105 was changed to a ser residue in *B. subtilis* 168 | This work |
| KO-SinR | BS168 derivate, deleting *sinR* | This work |
| Plasmid |  |  |
| P7Z6 | Harboring Amp [resistance](javascript:;) [gene](javascript:;) | Granted by Jiangnan University |
| Primers | Primers sequences (5′→3′) |  |
| KO-SinR-L-F | TTGAATCAGCCGGAGCTAAAGTCCC |  |
| KO-SinR-L-R | TAGAGGATCCCCGGGTTGTCATCACCTTCCTTGTGATATTAT |  |
| KO-p43c-L | GTGATGACAACCCGGGGATCCTCTAGA |  |
| KO-p43c-R | CTCAGGCAGTTCAAGCGAAAACATACCAC |  |
| KO-SinR-R-F | GCTTGAACTGCCTGAGCAGAGGC |  |
| KO-SinR-R-R | CAAAGGACAGCACCATGTCTACTTA |  |
| KO-ComA-L-F | GATGCAATCGGCATCAATCTG |  |
| KO-ComA-L-R | GATCCCCGGGTATGTTGGGGGGTGTAGAGA |  |
| KO-p43c-F | TACACCCCCCAACATACCCGGGGATCCTCTAGA |  |
| KO-p43c-R | GTAAAAGGGAGGAAAACGTTCAAGCGAAAACATACCACC |  |
| KO-ComA-R-F | TGTTTTCGCTTGAACGTTTTCCTCCCTTTTACTCACTCT |  |
| KO-ComA-R-R | GAAAACGTAACTTCAGAAATTGGGA |  |
| kinB-L-F | AGTTGTTCAGCCTCCAGTTT |  |
| kinB-L-R | CTAGAGGATCCCCGGGTTCGTGTGAAATCC |  |
| kinB-p43c-F | TTCACACGAACCCGGGGATCCTCTAGAGA |  |
| kinB-p43c-R | AGTCTTTTAGAATTTCCATGTGTACATTCCTCTCTTACCTATAATGG |  |
| kinB-R-F | AGAGAGGAATGTACACATGGAAATTCTAAAAGACTATCTTCTGC |  |
| kinB-R-R | TACGACACCATAAGGGAGCT |  |
| epsE-L-F | AGAAATTCTCCATCCCGAT |  |
| epsE-L-R | TCCCCGGGTACGCTTTTCTCCTTTGTATCCA |  |
| epsE-p43c-F | GAGAAAAGCGTACCCGGGGATCCTCTAG |  |
| epsE-p43c-R | CGGTCCTGAGTTCATGTGTACATTCCTCTCTTACCTATAATG |  |
| epsE-R-F | GAGGAATGTACACATGAACTCAGGACCGAAAGTT |  |
| epsE-R-R | GTAATCAGATAAAGGAAGCTTCAAG |  |
| tapA-L-F | TCAACGGATTCGGGAACAGA |  |
| tapA-L-R | ATCCCCGGGTATCTTACCTCCTGTAAAACACTG |  |
| tapA-p43c-F | AGGAGGTAAGATACCCGGGGATCCTCTAG |  |
| tapA-p43c-R | GTGAAACAATCGAAACATGTGTACATTCCTCTCTTACCTATAAT |  |
| tapA-R-F | AGAGGAATGTACACATGTTTCGATTGTTTCACAATCAG |  |
| tapA-R-R | CCCGCTTTCCTTCTGGTCTG |  |
| sinI-L-F | CGCCCTTTCTACTGGCATTG |  |
| sinI-L-R | CCCCGGGTGCAGTTTCTCCTCCTAAAATACTT |  |
| sinI-P7C6-R | CTAGAGGATCCCCGGGTGCAGTTTCT |  |
| sinI-P7C6-R | TTTGCATTCTTCATGTGTACATTCCTCTCTTACCTATAATGG |  |
| sinI-R-F | AGAGGAATGTACACATGAAGAATGCAAAACAAGAGCAC |  |
| sinI-R-R | CGGCGTGATGCTGTTAGTTT |  |
| E97K-F | CGAAAAAACAATTTCGTAAGTTTTTAGATTATCAAA |  |
| E97K-R | TTTGATAATCTAAAAACTTACGAAATTGTTTTTTCG |  |
| Y101L-F | CGTGAATTTTTAGATCTTCAAAAATGGAGAAAATCC |  |
| Y101L-R | GGATTTTCTCCATTTTTGAAGATCTAAAAATTCACG |  |
| W104K-F | TTTAGATTATCAAAAAAAGAGAAAATCCCAAAAAGAG |  |
| W104K-R | CTCTTTTTGGGATTTTCTCTTTTTTTGATAATCTAAA |  |
| R105S-F | AGATTATCAAAAATGGTCCAAATCCCAAAAAGAGGAG |  |
| R105S-R | CTCCTCTTTTTGGGATTTGGACCATTTTTGATAATCT |  |
| SinR^quad^-F | CAATTTCGTAAGTTTTTAGATCTTCAAAAAAAGTCAAAATCCC |  |
| SinR^quad^-R | GGGATTTGGACTTTTTTTGAAGATCTAAAAACTTACGAAATTG |  |
| SinR-F | TCCTCTACTACTCCTCTTTTTGGGATTTTCTCC |  |
| SinR-R | GTTCAAGCTTGATTGGCCAGCGTATTAAACAATACCG |  |
